# Supplementary material for: Rapid learning of a phonemic discrimination in the first hours of life
Source: Nat Hum Behav. 2022 Jun 2;6(8):1169–79. doi: 10.1038/s41562-022-01355-1 (PMC9391223; doi:10.1038/s41562-022-01355-1)
Supplement: Supplementary file 1 — Supplementary Figs. 1–3 and Tables 1–4. [file 41562_2022_1355_MOESM1_ESM.pdf]

nature portfolio

https://doi.org/10.1038/s41562-022-01355-1

# Supplementary information

# Rapid learning of a phonemic discrimination in the first hours of life

In the format provided by the authors and unedited

# Rapid learning of a phonemic discrimination in the first hours of life

Yan Jing Wu[1,#], Xinlin Hou[2,#], Cheng Peng[2], Wenwen Yu[3], Gary M. Oppenheim[4], Guillaume Thierry[4,5], Dandan Zhang[3,6,7]*

1 Faculty of Foreign Languages, Ningbo University, Ningbo 315211, China
2 Department of Pediatrics, Peking University First Hospital, Beijing 100034, China
3 School of Psychology, Shenzhen University, Shenzhen 518060, China
4 School of Psychology, Bangor University, Bangor, LL57 2AS, Wales, UK
5 Faculty of English, Adam Mickiewicz University, Poznań 61-180, Poland
6 Institute of Brain and Psychological Sciences, Sichuan Normal University, Chengdu 610066, China
7 Shenzhen-Hong Kong Institute of Brain Science, Shenzhen 518055, China

Running title: How neonates learn speech sounds on the first day

* Corresponding author

Dandan Zhang

zhangdd05@gmail.com

School of Psychology,

Shenzhen University

Shenzhen, China

#These authors contributed equally to the study.

Authors declare no conflict of interest in relation to the subject of this study.

# Supplementary material (additional methods and results)

## Figures

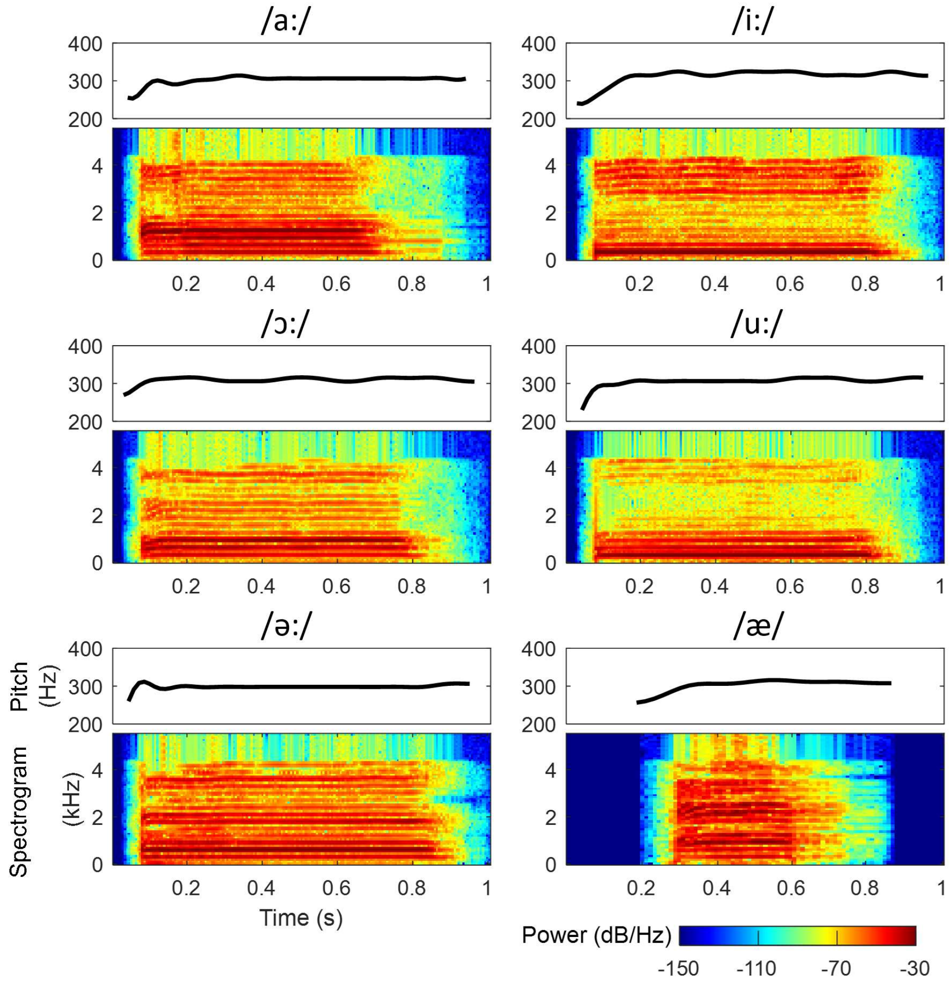


Supplementary Figure 1. The pitch (f0) contours (upper panel) and the spectrograms (lower panel) of the six vowels used in this study.

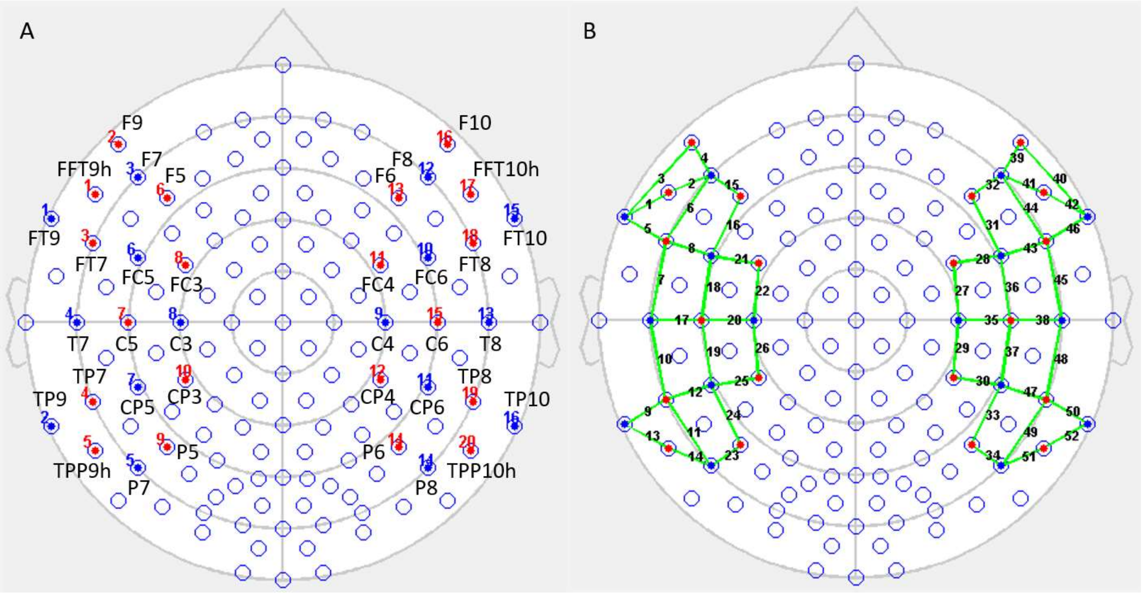


Supplementary Figure 2. Locations of optodes and channels with respect to the EEG 10/5 system. A, Twenty emitters were illustrated as red dots and sixteen detectors were illustrated as blue dots. B, The 20 × 16 optodes constitute 52 channels of interest (green lines).

## Tables

Supplementary Table 1. Estimates from the linear mixed effects regression analyses of mean amplitudes and peak latencies

| | Mean [HbO] amplitude | | | | | [HbO] peak latency | | | | |
|---|---|---|---|---|---|---|---|---|---|---|
| | beta | SE | df | *t* | *p* | beta | SE | df | *t* | *p* |
| (Intercept) | 0.844 | 0.010 | 111.3 | 85.33 | <.001 | 11.222 | 0.033 | 105.1 | 341.60 | <.001 |
| Group (Contrast 1: Passive control vs. mean(Active control, Experimental)) | -0.022 | 0.018 | 83.5 | -1.26 | 0.21 | -0.288 | 0.083 | 104.0 | -3.45 | <.001 |
| Group (Contrast 2: Active control vs. Experimental) | -0.030 | 0.019 | 73.5 | -1.55 | 0.12 | -0.552 | 0.132 | 78.4 | -4.20 | <.001 |
| Stimulus type (Backwards vs. Forwards) | 0.027 | 0.010 | 99.8 | 2.71 | 0.008 | -0.163 | 0.057 | 89.7 | -2.84 | 0.006 |
| Phase (Contrast 1: T0 vs. mean(T1, T2)) | -0.015 | 0.015 | 68.9 | -1.01 | 0.32 | -0.275 | 0.067 | 106.2 | -4.11 | <.001 |
| Phase (Contrast 2: T1 vs. T2) | 0.053 | 0.020 | 96.0 | 2.71 | 0.008 | -0.038 | 0.063 | 66.7 | -0.61 | 0.55 |
| Group (Contrast 1) x Stimulus type | 0.025 | 0.019 | 73.4 | 1.34 | 0.18 | -0.287 | 0.119 | 82.3 | -2.42 | 0.018 |
| Group (Contrast 2) x Stimulus type | 0.050 | 0.022 | 83.3 | 2.27 | 0.026 | -0.539 | 0.159 | 106.5 | -3.39 | <.001 |
| Group (Contrast 1) x Phase (Contrast 1) | 0.007 | 0.034 | 79.9 | 0.21 | 0.83 | -0.428 | 0.135 | 103.4 | -3.17 | 0.002 |
| Group (Contrast 1) x Phase (Contrast 2) | 0.084 | 0.040 | 84.9 | 2.08 | 0.04 | 0.038 | 0.143 | 82.0 | 0.27 | 0.79 |
| Group (Contrast 2) x Phase (Contrast 1) | -0.049 | 0.038 | 80.6 | -1.28 | 0.21 | -0.846 | 0.193 | 97.8 | -4.38 | <.001 |
| Group (Contrast 2) x Phase (Contrast 2) | 0.162 | 0.052 | 108.3 | 3.11 | 0.002 | -0.058 | 0.154 | 69.9 | -0.38 | 0.71 |
| Stimulus type x Phase (Contrast 1) | 0.012 | 0.022 | 78.5 | 0.56 | 0.58 | -0.224 | 0.084 | 91.7 | -2.68 | 0.009 |
| Stimulus type x Phase (Contrast 2) | 0.014 | 0.024 | 82.9 | 0.59 | 0.56 | -0.052 | 0.103 | 69.5 | -0.51 | 0.61 |
| Group (Contrast 1) x Stimulus type x Phase (Contrast 1) | 0.051 | 0.045 | 71.3 | 1.14 | 0.26 | -0.292 | 0.167 | 80,0 | -1.75 | 0.083 |
| Group (Contrast 1) x Stimulus type x Phase (Contrast 2) | 0.071 | 0.049 | 74.1 | 1.46 | 0.15 | 0.016 | 0.217 | 66.6 | 0.07 | 0.94 |
| Group (Contrast 2) x Stimulus type x Phase (Contrast 1) | 0.052 | 0.052 | 78.0 | 0.99 | 0.32 | **-0.569** | **0.209** | **95.0** | **-2.72** | **0.008** |
| Group (Contrast 2) x Stimulus type x Phase (Contrast 2) | **0.125** | **0.058** | **86.7** | **2.15** | **0.034** | -0.143 | 0.249 | 70.3 | -0.57 | 0.57 |

Beta: beta estimate, SE: standard error of the mean, df: degrees of freedom, t: t-value (two-tailed test), p = estimated p-value (uncorrected for multiple comparisons).

Supplementary Table 2. Sound materials used in training phase.

| Experimental group | | Active control group | |
|---|---|---|---|
| 1 | /ɑ:/ /i:/ /ɔ:/ /i:/ /ɔ:/ /ɑ:/ | 1 | /u:/ /ə:/ /æ/ /ə:/ /æ/ /u:/ |
| 2 | /ɑ:/ /i:/ /ɔ:/ /ɑ:/ /ɔ:/ /i:/ | 2 | /u:/ /ə:/ /æ/ /u:/ /æ/ /ə:/ |
| 3 | /ɑ:/ /ɔ:/ /i:/ /ɔ:/ /ɑ:/ /i:/ | 3 | /u:/ /æ/ /ə:/ /æ/ /u:/ /ə:/ |
| 4 | /ɑ:/ /ɔ:/ /i:/ /ɔ:/ /i:/ /ɑ:/ | 4 | /u:/ /æ/ /ə:/ /æ/ /ə:/ /u:/ |
| 5 | /i:/ /ɔ:/ /ɑ:/ /i:/ /ɑ:/ /ɔ:/ | 5 | /ə:/ /æ/ /u:/ /ə:/ /u:/ /æ/ |
| 6 | /i:/ /ɔ:/ /ɑ:/ /ɔ:/ /ɑ:/ /i:/ | 6 | /ə:/ /æ/ /u:/ /æ/ /u:/ /ə:/ |
| 7 | /i:/ /ɑ:/ /ɔ:/ /ɑ:/ /i:/ /ɔ:/ | 7 | /ə:/ /u:/ /æ/ /u:/ /ə:/ /æ/ |
| 8 | /i:/ /ɑ:/ /ɔ:/ /ɑ:/ /ɔ:/ /i:/ | 8 | /ə:/ /u:/ /æ/ /u:/ /æ/ /ə:/ |
| 9 | /ɔ:/ /ɑ:/ /i:/ /ɑ:/ /i:/ /ɔ:/ | 9 | /æ/ /u:/ /ə:/ /u:/ /ə:/ /æ/ |
| 10 | /ɔ:/ /ɑ:/ /i:/ /ɔ:/ /i:/ /ɑ:/ | 10 | /æ/ /u:/ /ə:/ /æ/ /ə:/ /u:/ |
| 11 | /ɔ:/ /i:/ /ɑ:/ /i:/ /ɔ:/ /ɑ:/ | 11 | /æ/ /ə:/ /u:/ /ə:/ /æ/ /u:/ |
| 12 | /ɔ:/ /i:/ /ɑ:/ /i:/ /ɑ:/ /ɔ:/ | 12 | /æ/ /ə:/ /u:/ /ə: /u:/ /æ/ |

Each sound string contains six vowels played forwardly or backward

Supplementary Table 3. Source-detector distance and spatial registration for NIRS channels using neonatal AAL template.

| # | Source-detector Distance (cm) | MNI coordinate | | | AAL (neonatal template) |
|---|---|---|---|---|---|
| | | x | y | z | |
| 1 | 2.0 | -37 | 10 | -12 | Temporal pole (superior) left |
| 2 | 1.8 | -39 | 14 | 7 | Inferior frontal gyrus (triangular) left |
| 3 | 3.5 | -36 | 11 | -12 | Temporal pole (superior) left |
| 4 | 2.0 | -37 | 18 | 10 | Inferior frontal gyrus (triangular) left |
| 5 | 2.5 | -41 | 3 | -1 | Superior temporal gyrus left |
| 6 | 2.0 | -40 | 9 | 15 | Inferior frontal gyrus (opercular) left |
| 7 | 2.2 | -42 | -6 | 5 | Superior temporal gyrus left |
| 8 | 2.5 | -42 | 2 | 12 | Postcentral gyrus left |
| 9 | 2.5 | -36 | -36 | -5 | Inferior temporal gyrus left |
| 10 | 2.8 | -42 | -25 | 9 | Superior temporal gyrus left |
| 11 | 2.0 | -38 | -48 | 6 | Middle temporal gyrus left |
| 12 | 2.3 | -40 | -43 | 18 | Angular gyrus left |
| 13 | 2.0 | -34 | -43 | -11 | Inferior occipital gyrus left |
| 14 | 2.0 | -34 | -52 | -1 | Middle occipital gyrus left |
| 15 | 2.0 | -36 | 19 | 17 | Inferior frontal gyrus (triangular) left |
| 16 | 2.5 | -36 | 12 | 25 | Inferior frontal gyrus (opercular) left |
| 17 | 2.2 | -44 | -15 | 18 | Supramarginal gyrus left |
| 18 | 2.0 | -44 | -7 | 28 | Postcentral gyrus left |
| 19 | 2.5 | -45 | -26 | 28 | Supramarginal gyrus left |
| 20 | 2.2 | -40 | -17 | 35 | Postcentral gyrus left |
| 21 | 2.2 | -37 | 3 | 31 | Precentral gyrus left |
| 22 | 2.5 | -33 | -7 | 41 | Precentral gyrus left |
| 23 | 2.5 | -38 | -49 | 19 | Angular gyrus left |
| 24 | 2.5 | -40 | -44 | 25 | Angular gyrus left |
| 25 | 2.5 | -40 | -37 | 30 | Inferior parietal lobule left |
| 26 | 2.0 | -36 | -32 | 40 | Inferior parietal lobule left |
| 27 | 2.5 | 30 | -8 | 40 | Precentral gyrus right |
| 28 | 2.2 | 33 | 1 | 35 | Middle frontal gyrus right |
| 29 | 2.0 | 36 | -29 | 40 | Superior parietal gyrus right |
| 30 | 2.5 | 37 | -38 | 35 | Inferior parietal lobule right |
| 31 | 2.5 | 36 | 10 | 26 | Inferior frontal gyrus (opercular) right |
| 32 | 2.0 | 34 | 17 | 20 | Middle frontal gyrus right |
| 33 | 2.5 | 37 | -45 | 28 | Angular gyrus right |
| 34 | 2.5 | 35 | -52 | 22 | Angular gyrus right |
| 35 | 2.2 | 37 | -18 | 38 | Postcentral gyrus right |
| 36 | 2.0 | 39 | -8 | 32 | Precentral gyrus right |
| 37 | 2.5 | 41 | -28 | 30 | Supramarginal gyrus right |
| 38 | 2.2 | 41 | -16 | 22 | Supramarginal gyrus right |
| 39 | 2.0 | 32 | 14 | 10 | Inferior frontal gyrus (triangular) right |
| 40 | 3.5 | 35 | 7 | -11 | Temporal pole (superior) right |
| 41 | 1.8 | 35 | 17 | 10 | Inferior frontal gyrus (triangular) right |
| 42 | 2.0 | 35 | 10 | -11 | Temporal pole (superior) right |
| 43 | 2.5 | 38 | 4 | 12 | Inferior frontal gyrus (opercular) right |
| 44 | 2.0 | 36 | 10 | 21 | Inferior frontal gyrus (opercular) right |
| 45 | 2.2 | 38 | -8 | 5 | Superior temporal gyrus right |
| 46 | 2.5 | 36 | 7 | -4 | Temporal pole (superior) right |
| 47 | 2.3 | 38 | -46 | 22 | Angular gyrus right |
| 48 | 2.8 | 38 | -24 | 12 | Superior temporal gyrus right |
| 49 | 2.0 | 35 | -51 | 11 | Middle temporal gyrus right |
| 50 | 2.5 | 36 | -41 | 1 | Middle temporal gyrus right |
| 51 | 2.0 | 31 | -56 | 2 | Middle occipital gyrus right |
| 52 | 2.0 | 31 | -46 | -10 | Inferior temporal gyrus right |

Supplementary Table 4. Eye/body characteristics and heart/respiration rates of the neonates during T0, T1, T2, training, and consolidation phases.

| Characteristic | Group | T0 (8 min) | | Training (5 h) | | T1 (8 min) | | Consolidation (2 h) | | T2 (8 min) | |
|---|---|---|---|---|---|---|---|---|---|---|---|
| | | descriptive | *p* | descriptive | *p* | descriptive | *p* | descriptive | *p* | descriptive | *p* |
| Eye opening duration (%) | experimental | 13.3 ± 10.5 | | 5.8 ± 3.7 | | 15.2 ± 13.8 | | 0.3 ± 0.5 | | 12.7 ± 14.0 | |
| | active control | 15.6 ± 11.5 | 0.654 | 6.4 ± 4.3 | 0.749 | 11.8 ± 13.8 | 0.740 | 0.4 ± 0.7 | 0.790 | 11.3 ± 13.0 | .862 |
| | passive control | 16.7 ± 15.1 | | 5.5 ± 3.1 | | 13.3 ± 16.2 | | 0.4 ± 0.8 | | 13.6 ± 14.9 | |
| Eye opening count | experimental | 1.3 ± 0.8 | | 6.0 ± 3.6 | | 1.1 ± 0.8 | | 0.5 ± 0.6 | | 0.9 ± 0.9 | |
| | active control | 1.3 ± 0.8 | 0.944 | 6.7 ± 4.2 | 0.708 | 1.0 ± 0.7 | 0.842 | 0.6 ± 0.7 | 0.742 | 0.9 ± 0.9 | 0.865 |
| | passive control | 1.2 ± 0.9 | | 5.8 ± 3.8 | | 1.1 ± 0.9 | | 0.7 ± 0.8 | | 1.0 ± 1.0 | |
| Body movement duration (%) | experimental | 20.0 ± 13.0 | | 5.7 ± 2.1 | | 18.8 ± 13.0 | | 6.7 ± 2.1 | | 14.1 ± 13.0 | |
| | active control | 18.0 ± 14.4 | 0.851 | 5.5 ± 2.2 | 0.943 | 19.9 ± 14.8 | 0.779 | 9.3 ± 2.2 | 0.665 | 16.9 ± 15.1 | 0.719 |
| | passive control | 20.2 ± 16.7 | | 5.8 ± 2.9 | | 17.0 ± 13.3 | | 7.6 ± 2.9 | | 17.2 ± 13.6 | |
| Body movement count | experimental | 1.8 ± 1.2 | | 6.2 ± 3.3 | | 1.6 ± 1.1 | | 0.7 ± 0.7 | | 1.4 ± 1.2 | |
| | active control | 1.8 ± 1.2 | 0.961 | 6.1 ± 3.1 | 0.786 | 1.5 ± 1.1 | 0.820 | 1.0 ± 0.9 | 0.509 | 1.6 ± 1.4 | 0.698 |
| | passive control | 1.7 ± 1.3 | | 6.7 ± 3.1 | | 1.4 ± 1.1 | | 0.8 ± 0.8 | | 1.8 ± 1.4 | |
| Cry during (%) | experimental | 0.4 ± 1.4 | | 3.5 ± 5.4 | | 0.6 ± 2.2 | | 0.0 ± 1.9 | | 0.5 ± 1.7 | |
| | active control | 0.6 ± 2.6 | 0.915 | 4.4 ± 6.0 | 0.847 | 0.3 ± 1.3 | 0.840 | 0.1 ± 3.8 | 0.923 | 0.5 ± 2.1 | 0.990 |
| | passive control | 0.5 ± 1.8 | | 3.7 ± 4.4 | | 0.4 ± 1.4 | | 0.1 ± 4.5 | | 0.6 ± 1.9 | |
| Cry count | experimental | 0.1 ± 0.3 | | 1.5 ± 0.7 | | 0.1 ± 0.3 | | 0.1 ± 0.3 | | 0.1 ± 0.3 | |
| | active control | 0.1 ± 0.3 | 0.996 | 1.7 ± 0.7 | 0.588 | 0.0 ± 0.2 | 0.776 | 0.1 ± 0.3 | 0.845 | 0.0 ± 0.3 | 0.996 |
| | passive control | 0.1 ± 0.3 | | 1.7 ± 0.6 | | 0.1 ± 0.3 | | 0.0 ± 0.2 | | 0.1 ± 0.3 | |
| Hear rate (counts/min) | experimental | 139.1 ± 4.3 | | 139.7 ± 5.1 | | 140.6 ± 3.4 | | 139.1 ± 5.1 | | 141.0 ± 4.5 | |
| | active control | 140.9 ± 4.8 | 0.325 | 138.6 ± 4.6 | 0.702 | 141.6 ± 4.0 | 0.698 | 138.7 ± 4.3 | 0.957 | 140.2 ± 4.6 | 0.749 |
| | passive control | 139.8 ± 4.4 | | 138.5 ± 5.8 | | 141.0 ± 4.6 | | 138.9 ± 4.0 | | 140.1 ± 4.4 | |
| Respiration rate (counts/min) | experimental | 46.6 ± 3.0 | | 43.2 ± 4.4 | | 45.4 ± 3.1 | | 42.6 ± 5.2 | | 46.5 ± 3.3 | |
| | active control | 46.1 ± 3.4 | 0.843 | 41.4 ± 5.1 | 0.327 | 44.6 ± 3.7 | 0.505 | 40.6 ± 5.7 | 0.364 | 45.3 ± 4.5 | 0.552 |
| | passive control | 46.5 ± 3.3 | | 41.3 ± 4.2 | | 45.9 ± 3.6 | | 40.8 ± 4.2 | | 46.2 ± 4.0 | |

The characteristics of count are defined as how many times of events during the whole phase. If neonates opened eyes, moved bodies, or cried twice within an interval of 1 min, the count was only one. One-way ANOVAs were performed on each measure in different phases to examine whether there was difference across the three groups. The *p* values were reported.
