# Supplementary material for: Rapid learning of a phonemic discrimination in the first hours of life
Source: Nat Hum Behav. 2022 Jun 2;6(8):1169–79. doi: 10.1038/s41562-022-01355-1 (PMC9391223; doi:10.1038/s41562-022-01355-1)
Supplement: Supplementary file 2 — Reporting Summary [file 41562_2022_1355_MOESM2_ESM.pdf]

nature portfolio

Corresponding author(s): Dandan Zhang

Last updated by author(s): Mar 31, 2022

# Reporting Summary

Nature Portfolio wishes to improve the reproducibility of the work that we publish. This form provides structure for consistency and transparency in reporting. For further information on Nature Portfolio policies, see our Editorial Policies and the Editorial Policy Checklist.

## Statistics

For all statistical analyses, confirm that the following items are present in the figure legend, table legend, main text, or Methods section.

| n/a | Confirmed | |
|---|---|---|
| ☐ | ☒ | The exact sample size (*n*) for each experimental group/condition, given as a discrete number and unit of measurement |
| ☐ | ☒ | A statement on whether measurements were taken from distinct samples or whether the same sample was measured repeatedly |
| ☐ | ☒ | The statistical test(s) used AND whether they are one- or two-sided<br>*Only common tests should be described solely by name; describe more complex techniques in the Methods section.* |
| ☒ | ☐ | A description of all covariates tested |
| ☐ | ☒ | A description of any assumptions or corrections, such as tests of normality and adjustment for multiple comparisons |
| ☐ | ☒ | A full description of the statistical parameters including central tendency (e.g. means) or other basic estimates (e.g. regression coefficient) AND variation (e.g. standard deviation) or associated estimates of uncertainty (e.g. confidence intervals) |
| ☐ | ☒ | For null hypothesis testing, the test statistic (e.g. *F*, *t*, *r*) with confidence intervals, effect sizes, degrees of freedom and *P* value noted<br>*Give P values as exact values whenever suitable.* |
| ☒ | ☐ | For Bayesian analysis, information on the choice of priors and Markov chain Monte Carlo settings |
| ☒ | ☐ | For hierarchical and complex designs, identification of the appropriate level for tests and full reporting of outcomes |
| ☐ | ☒ | Estimates of effect sizes (e.g. Cohen's *d*, Pearson's *r*), indicating how they were calculated |

*Our web collection on statistics for biologists contains articles on many of the points above.*

## Software and code

Policy information about availability of computer code

| | |
|---|---|
| Data collection | NIRS Data was collected  using the NirSmart system (Danyang Huichuang, China). Sound materials were edited using Cool Edit Pro 2.1 (Syntrillium Software Corp., AZ, USA). |
| Data analysis | Pre-processing and statistical analyses were conducted in Matlab (v2021a, the Mathworks, Inc., Natick, USA). NIRS Data analysis was performed using the NirSmart system (Danyang Huichuang, China).  Linear mixed effects models were conducted using the R package lme4 (Bates et al., 2015). |

For manuscripts utilizing custom algorithms or software that are central to the research but not yet described in published literature, software must be made available to editors and reviewers. We strongly encourage code deposition in a community repository (e.g. GitHub). See the Nature Portfolio guidelines for submitting code & software for further information.

## Data

Policy information about availability of data

All manuscripts must include a data availability statement. This statement should provide the following information, where applicable:

- Accession codes, unique identifiers, or web links for publicly available datasets
- A description of any restrictions on data availability
- For clinical datasets or third party data, please ensure that the statement adheres to our policy

The data for linear mixed effects regression as well as a full report of the NIRS results are upload as supplementary files. Additional data would be available upon reasonable request and with approval of the School of Psychology, Shenzhen University. More information on making this request can be obtained from the corresponding author, D. Zhang (zhangdd05@gmail.com).

# Field-specific reporting

Please select the one below that is the best fit for your research. If you are not sure, read the appropriate sections before making your selection.

☒ Life sciences ☐ Behavioural & social sciences ☐ Ecological, evolutionary & environmental sciences

For a reference copy of the document with all sections, see nature.com/documents/nr-reporting-summary-flat.pdf

# Life sciences study design

All studies must disclose on these points even when the disclosure is negative.

**Sample size**

Seventy-five healthy full-term neonates were randomly assigned into the experimental group (n = 25), the active control group (n = 25), and the passive control group (n = 25). Since data collection in healthy, full-term neonates has practical limits, the sample size was based on previous studies on neonates with similar research objectives (e.g., Benavides-Varela et al., PNAS, 2012; Cabrera & Gervain, Sci Adv, 2020; Gervain et al., PNAS, 2008; Gómez et al., PNAS, 2014; May et al., Dev Sci, 2018; Peña et al., PNAS, 2003; Perani et al., PNAS, 2011).

**Data exclusions**

Neonates who started crying during the recording were excluded from the analyses (i.e., 3 from the experimental group, 2 from the active control group, and 4 from the passive control group).

**Replication**

Results in the current study are not replicated because the study was conducted on neonates which requires a substantial period of time to collect data and, therefore, to replicate the current study. However, details of the experiments (i.e.,procedure and stimuli) and data analysis are provided in the manuscript, allowing future replications of this study.

**Randomization**

Participants were randomly assigned into the experimental and control groups.

**Blinding**

The investigator who collected the data was blinded to group allocation during data collection and was debriefed with the purpose of the study afterwards. The researcher who analyzed the data was also blind to the conditions of the experiment, as the conditions were coded during data analysis.

# Reporting for specific materials, systems and methods

We require information from authors about some types of materials, experimental systems and methods used in many studies. Here, indicate whether each material, system or method listed is relevant to your study. If you are not sure if a list item applies to your research, read the appropriate section before selecting a response.

## Materials & experimental systems

| n/a | Involved in the study | |
|---|---|---|
| ☒ | ☐ | Antibodies |
| ☒ | ☐ | Eukaryotic cell lines |
| ☒ | ☐ | Palaeontology and archaeology |
| ☒ | ☐ | Animals and other organisms |
| ☐ | ☒ | Human research participants |
| ☒ | ☐ | Clinical data |
| ☒ | ☐ | Dual use research of concern |

## Methods

| n/a | Involved in the study | |
|---|---|---|
| ☒ | ☐ | ChIP-seq |
| ☒ | ☐ | Flow cytometry |
| ☒ | ☐ | MRI-based neuroimaging |

## Human research participants

Policy information about studies involving human research participants

**Population characteristics**

Seventy-five healthy, full-term neonates (38 boys; gestational age: 38 to 41 weeks, mean = 39.0 ± 0.7 weeks) within 1 to 3 hours (mean = 2.1 ± 0.4 hours) of birth were recruited in the study.

**Recruitment**

Recruitment advertisement was posted at the entrance to the obstetrics department of Peking University First Hospital. Parents who were willing to take part in the study contacted the experimenter and their information was recorded. Among these recorded neonates, the ones who qualified the requirements of the study (see Participants in the Methods section) were recruited on the first day after birth. Written consent was obtained from parents prior to data collection.

**Ethics oversight**

This study was approved by the Ethical Committee of Peking University First Hospital.

Note that full information on the approval of the study protocol must also be provided in the manuscript.
